# Youth access and utilization of HIV services in Homa Bay, Kenya: protocol for a mixed-methods study

**DOI:** 10.3389/frhs.2026.1892411

**Published:** 2026-10-02

**Authors:** Lawrence Ojala, Wilson Manyasa, Mabel Nangami, Nyawira Gitahi, Leslie A. Enane

**Affiliations:** 1 School of Public Health, Moi University College of Health Sciences, Eldoret, Kenya; 2 Department of Sustainable Livelihood, Shining Hope for the Communities, Nairobi, Kenya; 3 Department of Health Management and Health Policy, School of Public Health, Moi University College of Health Sciences, Eldoret, Kenya; 4 Kenya Medical Research Institute, University of Toronto, Toronto, ON, Canada; 5 The Ryan White Center for Pediatric Infectious Disease and Global Health, Department of Pediatrics, Indiana University School of Medicine, Indianapolis, IN, United States

**Keywords:** adolescents, healthcare access, HIV services, Homa Bay, Kenya, mixed methods, service utilization, youth

## Abstract

**Background:**

Youth aged 15–24 years continue to experience substantial HIV-related vulnerability, particularly in sub-Saharan Africa. Although HIV prevention, testing, treatment and support services have expanded, barriers related to knowledge, stigma, confidentiality, affordability, geographic accessibility, provider attitudes and the organization of health services continue to affect young people's engagement with HIV services. Homa Bay County has one of the highest HIV burdens in Kenya, yet evidence on the factors influencing youth access to and utilization of HIV services in the county remains limited.

**Objective:**

This study aims to assess access to and utilization of HIV services among youth aged 15–24 years in Homa Bay Town Sub-County, Kenya, and to examine individual, household, community and health-system factors associated with utilization.

**Methods:**

A mixed-methods cross-sectional study will be conducted among youth aged 15–24 years residing in Homa Bay Town Sub-County. The quantitative component will target 400 youth recruited through a multistage sampling approach involving ward-based stratification, selection of community-based clusters, and participant selection within selected clusters. Quantitative data will be collected using a structured researcher-administered questionnaire. The qualitative component will comprise focus group discussions with youth and key informant interviews with healthcare providers, peer educators and community gatekeepers. Quantitative data will be analysed using R. Descriptive statistics and bivariate analyses will be followed by multivariable logistic regression to identify factors independently associated with HIV service utilization. Qualitative data will be analysed thematically. Findings from the two components will be integrated using narrative synthesis and thematic matrices.

**Expected contribution:**

The study will generate context-specific evidence on individual, social and health-system factors influencing youth HIV service utilization in Homa Bay Town Sub-County. The findings will inform the design and strengthening of youth-friendly HIV services and contribute to implementation strategies aimed at reducing barriers to HIV prevention, testing and care.

## Introduction

### Background

Globally, over 8% of people living with HIV are youth, among whom nearly 80% reside in sub-Saharan Africa (1, 2). Many youths in sub-Saharan Africa are unaware of their HIV status, though knowing their status is critical for starting antiretroviral therapy (ART) to improve their health and avoid preventable morbidity and mortality (3). HIV testing and counselling is essential for both treatment and prevention efforts (4).

**Table 1:** Key informant interviews.

Key Informant Category	Estimated Number
Health care providers	4–6
Peer Educators	4–6
Community Gatekeepers (e.g., chiefs, religious leaders, youth leaders)	4–6

The total number of KIIs is expected to range between 12 and 18 participants across the wards, or until information saturation is achieved.

**Table 2:** Study variables.

Variable Category	Specific Variables	Type	Measurement/Description
Demographic and socioeconomic characteristics	Age, sex, educational level, employment status, household socioeconomic status	Quantitative	Age in completed years; sex as reported by participant; highest level of education attained; employment/student status; socioeconomic status measured using predefined household indicators
HIV knowledge	Knowledge of HIV transmission, prevention, testing and treatment	Quantitative	Composite score derived from structured knowledge questions. Correct responses will be coded and summed, with higher scores indicating greater HIV knowledge
Access to HIV services	Physical availability, distance, affordability, convenience, operating hours, waiting time, perceived ease of obtaining services	Quantitative	Access will be measured using structured questions assessing whether participants can obtain HIV services when needed and perceived barriers to accessing services. The primary access outcome will be categorized as adequate access versus inadequate access based on the predefined study scoring criteria
HIV service utilization	HIV testing, HIV counselling, prevention services and participation in youth-focused HIV programmes	Quantitative	Self-reported use of HIV services within the specified recall period. The primary utilization outcome will be categorized as utilized versus not utilized based on whether the participant reported using at least one specified HIV service during the defined recall period
Individual/user-side factors	HIV knowledge, perceived risk, stigma, beliefs, perceived benefits, confidentiality concerns and previous experience with health services	Quantitative/Qualitative	Measured using structured questionnaire items and explored qualitatively through FGDs and KIIs
Interpersonal/community factors	Family support, peer influence, social norms, community stigma, religious and cultural beliefs	Quantitative/Qualitative	Structured questionnaire items and narrative responses from FGDs and KIIs
Health-system/ institutional factors	Distance to facility, provider attitudes, confidentiality, waiting time, availability of commodities and services, operating hours, youth-friendly spaces and perceived quality of care	Quantitative/Qualitative	Quantitative items where applicable and qualitative narratives from participants, providers and key informants
Experiences and perceptions	Experiences with accessing or using HIV services; perceptions of availability, accessibility, acceptability and quality	Qualitative	Narrative responses obtained through FGDs and KIIs
Barriers and facilitators	Stigma, cost, distance, transportation, confidentiality concerns, cultural norms, provider attitudes, service availability and other identified factors	Qualitative	Themes and subthemes generated from FGD and KII transcripts

**Table 3:** Data analysis plan summary.

Objective	Primary variables/outcome	Data source	Planned analysis
Determine HIV service utilization among youth	Use of at least one HIV-related service; service-specific utilization	Quantitative	Frequencies, percentages and descriptive statistics
Examine individual factors associated with HIV service utilization	Age, sex, education, school-going status, HIV knowledge, perceived HIV risk and stigma	Quantitative	Bivariate analysis and multivariable logistic regression
Examine household and interpersonal factors associated with HIV service utilization	Socioeconomic circumstances, family support and peer influence	Quantitative/qualitative	Bivariate analysis, multivariable logistic regression and thematic analysis
Examine community factors associated with HIV service utilization	Community stigma, social norms, cultural and religious beliefs	Quantitative/qualitative	Bivariate analysis, multivariable logistic regression and thematic analysis
Examine health-system factors associated with HIV service utilization	Distance, affordability, operating hours, waiting time, privacy, confidentiality, provider attitudes and youth-friendly service characteristics	Quantitative/qualitative	Bivariate analysis, multivariable logistic regression and thematic analysis
Explore barriers and facilitators influencing HIV service utilization	Participant experiences, perceptions and reported barriers/facilitators	FGDs/KIIs	Thematic analysis using deductive and inductive coding
Integrate quantitative and qualitative findings	Areas of convergence, complementarity and divergence	Quantitative and qualitative components	Narrative synthesis and thematic matrices

HIV service programs should protect youth privacy, confidentiality, respect, and ensure informed consent. Youth-friendly services (YFS) are designed to meet the healthcare needs of young people, with an emphasis on accessibility, acceptability, equity, appropriateness, and effectiveness (5). HIV testing access and uptake vary across countries and communities in sub-Saharan Africa, with significant coverage gaps. Although over 77% of people diagnosed with HIV are on ART, the majority remain undiagnosed (6). Uptake of HIV services among Kenyan youth remains low, shaped by healthcare quality concerns and access barriers (7).

According to the 2025 National Syndemic and Disease Control Council estimates, about 1.32 million people in Kenya are living with HIV, with youth aged 15–24 years contributing approximately 132,018 cases (10%) (8). In Homa Bay County, where HIV prevalence is among the highest nationally (9), 104,317 people are living with HIV, including 12,180 youths (11.7%), ranking the county second after Nairobi in youth HIV burden (10).

New infections highlight the severity of the problem. Kenya recorded 20,105 new HIV infections in 2025, with 6,362 (31.6%) among youth. In Homa Bay, new infections reached 1,180, with 469 (39.7%) among youth. Mortality patterns show a similar trend: nationally, HIV-related deaths total 21,009, of which 1,972 (9.4%) are youth, while Homa Bay records 143 HIV-related deaths in youth, the highest in the country (10).

In Homa Bay County, where HIV prevalence is nearly 4 times higher than the national figure, youth aged 15–24 face multiple barriers to accessing and utilizing HIV services. Limited knowledge, concerns about privacy, low uptake of HIV testing, and poor provider relationships with the users (youth) hinder HIV service uptake (11). While youth-friendly services are recognized as essential for prevention, testing, and treatment, their accessibility and utilization remain inadequately understood in Homa Bay (12).

### Individual, household and community determinants

Individuals who perceive themselves at higher risk are more likely to seek preventive services such as condom use and HIV testing (13). At the household level, socioeconomic status strongly influences access and utilization of HIV services, with low-income families facing greater barriers (14). At the community level, peer-led interventions, support groups, and broader initiatives play a key role in improving youth-friendly HIV service uptake, while integrating culturally sensitive counselling, psychosocial support, and mental health care helps build resilience and promote positive health outcomes (15).

One limitation of many health systems strengthening frameworks is the inadequate consideration of socio-cultural factors that influence healthcare access and utilization. The missing link so far is the absence of user perspectives and participation of users as represented by the people, households, or communities in the delivery of healthcare services.

### Health-system and institutional determinants

Integrating HIV with other health services increases access and use among young people (16, 17). By contrast, long waiting times, lack of privacy, unfriendly staff, and stigma make services unacceptable to many youths. Youth from marginalized groups, such as street-involved youth, face particular inequities despite services in care access, raising issues for healthcare equity, including those related to costs of services (18).

Low uptake of HIV services among Kenyan youth is strongly linked to systemic gaps in the healthcare system, including shortages of trained providers, confidentiality concerns, and inequitable service delivery (19).

### Problem statement

Globally, youth continue to experience a disproportionate burden of HIV infection and AIDS-related mortality. Young people account for a substantial proportion of new HIV infections globally, while adolescents and young adults continue to face barriers to accessing HIV prevention, testing, treatment, and other HIV-related services (20). Although numerous interventions have been implemented to improve HIV service delivery for youths, uptake and utilization remain suboptimal, particularly in sub-Saharan Africa (21). This suggests that the availability of HIV services alone may not be sufficient to ensure that youth's access and consistently utilize them.

Evidence from Kenya indicates that utilization of HIV services among youths is influenced by multiple and interconnected factors. Previous studies have identified individual-level barriers such as inadequate knowledge, low perceived risk of HIV infection, fear of testing and diagnosis, and concerns about stigma. Interpersonal and community factors, including parental or partner influence, peer norms, stigma, and concerns about confidentiality, have also been reported to affect young people's willingness to seek HIV services (22). At the health-system level, studies have identified concerns related to provider attitudes, long waiting times, inconvenient operating hours, lack of privacy, distance and transport costs, and limited availability of youth-friendly services (23). These findings demonstrate that youth HIV service utilization is shaped not only by individual behaviour but also by the social and health-system environments in which young people seek care.

However, important gaps remain in the Kenyan evidence. Much of the existing research has examined individual barriers or specific HIV services, such as HIV testing, rather than comprehensively examining how individual, interpersonal, community, and health-system factors interact to influence access to and utilization of a broad range of HIV services among youths. Furthermore, evidence generated at national or other county levels may not adequately reflect the realities of youths living in Homa Bay County, where the HIV epidemic is particularly pronounced. Consequently, there is limited context-specific evidence on the factors that facilitate or constrain young people's access to and utilization of HIV services in Homa Bay, including how service characteristics, provider experiences, social norms, perceived stigma, and individual characteristics combine to influence service use.

The need for context-specific evidence is particularly important in Homa Bay County, which continues to experience a substantial HIV burden. Youths reportedly account for approximately 38% of new HIV infections in the county, which also has one of the highest HIV burdens and mortality rates in Kenya (24). Despite substantial investment by government and non-governmental organizations in HIV prevention, testing, treatment, and other interventions in Homa Bay, persistent barriers to HIV service access and utilization among youths remain. Existing programs have largely emphasized biomedical approaches, while comparatively less attention has been given to addressing the behavioural, social, community, and health-system factors that may prevent young people from accessing and consistently using available services (25). The persistence of these barriers despite ongoing HIV programming suggests that existing interventions may not have sufficiently addressed the diverse and context-specific needs and preferences of youths.

Therefore, the critical knowledge gap is not simply whether HIV services are available in Homa Bay, but why youths who need these services may not access or utilize them and which factors across the individual, interpersonal, community, and health-system levels are most influential. Without such context-specific evidence, HIV programs may continue to emphasize service availability without adequately addressing the barriers that shape actual service use. This study therefore seeks to assess the factors influencing access to and utilization of HIV services among youths in Homa Bay Town Sub-County, providing evidence to inform more responsive, youth-centred, and contextually appropriate HIV interventions and service delivery approaches.

### Justification

HIV prevention and treatment interventions are more likely to be effective when they address biomedical, behavioural, social, and structural determinants simultaneously.

Understanding whether youth can access HIV services is distinct from determining whether they actually utilize those services. A service may be physically available but remain underutilized because of stigma, lack of confidentiality, inconvenient operating hours, provider attitudes, cost, or distance.

This study will therefore distinguish between access-related conditions and actual service utilization. Quantitative data will provide estimates of utilization and its associated factors, while qualitative data will explain how and why individual, social and institutional barriers influence young people's decisions.

The findings are expected to support the design of more responsive youth-friendly HIV services in Homa Bay Town Sub-County.

### Research question

#### Primary research question

What individual, household, community and health-system factors influence access to and utilization of HIV services among youth aged 15–24 years in Homa Bay Town Sub-County, Kenya?

### Secondary questions

- What proportion of youth utilize HIV-related services?
- What individual, household, and community factors influence utilization?
- What health-system characteristics influence utilization?
- What barriers and facilitators do youth and stakeholders perceive?

### Broad objective

To assess access to and utilization of HIV services among youth aged 15–24 years in Homa Bay Town Sub-County, Kenya, and to examine individual, household, community, and health-system factors associated with utilization.

#### Specific objectives

- To determine the level and pattern of HIV service utilization among youth aged 15–24 years.
- To examine individual, household, and community factors associated with HIV service utilization among youth.
- To assess health-system factors associated with HIV service utilization among youth.
- To explore youth and stakeholder perspectives on barriers and facilitators to accessing and utilizing HIV services.

### Significance of the study

Evidence shows that combining biomedical, structural, and behavioural interventions at multiple levels enhances HIV prevention (26). Involving end-users further improves uptake of testing and preventive services (27).

Homa Bay County, despite being among the counties with the highest prevalence nationally, faces a growing youth HIV burden, yet HIV service uptake remains low, with only about half of young people willing to use available services (28). Understanding the diverse barriers limiting access and utilization is therefore critical to designing targeted, youth-friendly interventions. Findings from this study will inform policy, strengthen service delivery, and improve health outcomes among this vulnerable group.

### Operational definitions

**Youth:** refers to individuals aged 15–24 years at the time of recruitment.

**Access to HIV services:** refers to the extent to which young people are able to obtain HIV-related services when needed.

Access will be considered multidimensional and will include:

- **Geographic accessibility:** distance and ability to reach a service point;
- **Financial accessibility:** whether services are free or affordable and associated transport costs;
- **Organizational accessibility:** operating hours, waiting time and convenience;
- **Acceptability:** provider friendliness, privacy, confidentiality and youth-friendliness; and
- **Availability:** whether relevant HIV services and commodities are available when required.

**HIV service utilization:** refers to the actual use of at least one HIV-related service by a participant.

For the primary quantitative analysis, utilization will be coded:

- **1 =** used at least one HIV-related service
- **0 =** has never used any HIV-related service

The study will assess utilization of services including:

- HIV testing and counselling;
- HIV prevention education and awareness;
- condom services;
- PrEP;
- PEP;
- ART-related services;
- adherence support; and
- youth HIV support activities where available.

**Youth-friendly HIV services:** refer to services that are accessible, acceptable, appropriate, equitable, confidential and responsive to young people's needs, including respectful provider-client relationships, privacy, convenient service arrangements and appropriate communication.

**HIV knowledge:** refer to participants’ self-reported knowledge concerning HIV transmission, prevention, testing, treatment and available services.

**Stigma:** negative beliefs, anticipated judgment, discrimination or social concerns that may discourage youth from seeking or using HIV services.

### Operationalisation of access and utilisation

**Primary outcome**—is the overall HIV service utilisation among youths age 15–24 years, defined as self-reported use of at least one HIV-related service during the specified recall period and coded as 1=utilised and 0=not utilised.

**Secondary outcomes**—It comprises service-specific utilisation and access to HIV services. Service-specific utilisation includes HIV testing and counselling, HIV education and awareness, condom services, PrEP, PEP, ART-related services, and adherence/support services, each measured as reported use and summarized using frequencies and proportions. Access will be measured using structured questionnaire items assessing distance, affordability, operating hours, waiting time, confidentiality, provider-related factors, and availability of services and commodities. These indicators will be used to characterize access barriers and facilitators and assess their association with the primary utilisation outcome.

Qualitative data from Focus Group Discussions and Key Informant Interviews will complement the quantitative findings by explaining perceived barriers and facilitators influencing access and utilisation.

### Conceptual framework

The conceptual framework integrates the Social Ecological Model (SEM) with the WHO youth-friendly health services framework to explain factors influencing HIV service utilization among youths. It considers interconnected influences at the demographic, individual, interpersonal, community, and health-system levels, including personal characteristics, social support, community norms, stigma, and service accessibility (29–31).

The primary outcome is HIV service utilization, categorized as use or non-use of at least one HIV service. The framework further considers utilization of specific services, including HTC, HIV education, condom services, PrEP, PEP, ART-related, and adherence/support services, as illustrated in Figure 1.

**Figure 1:**
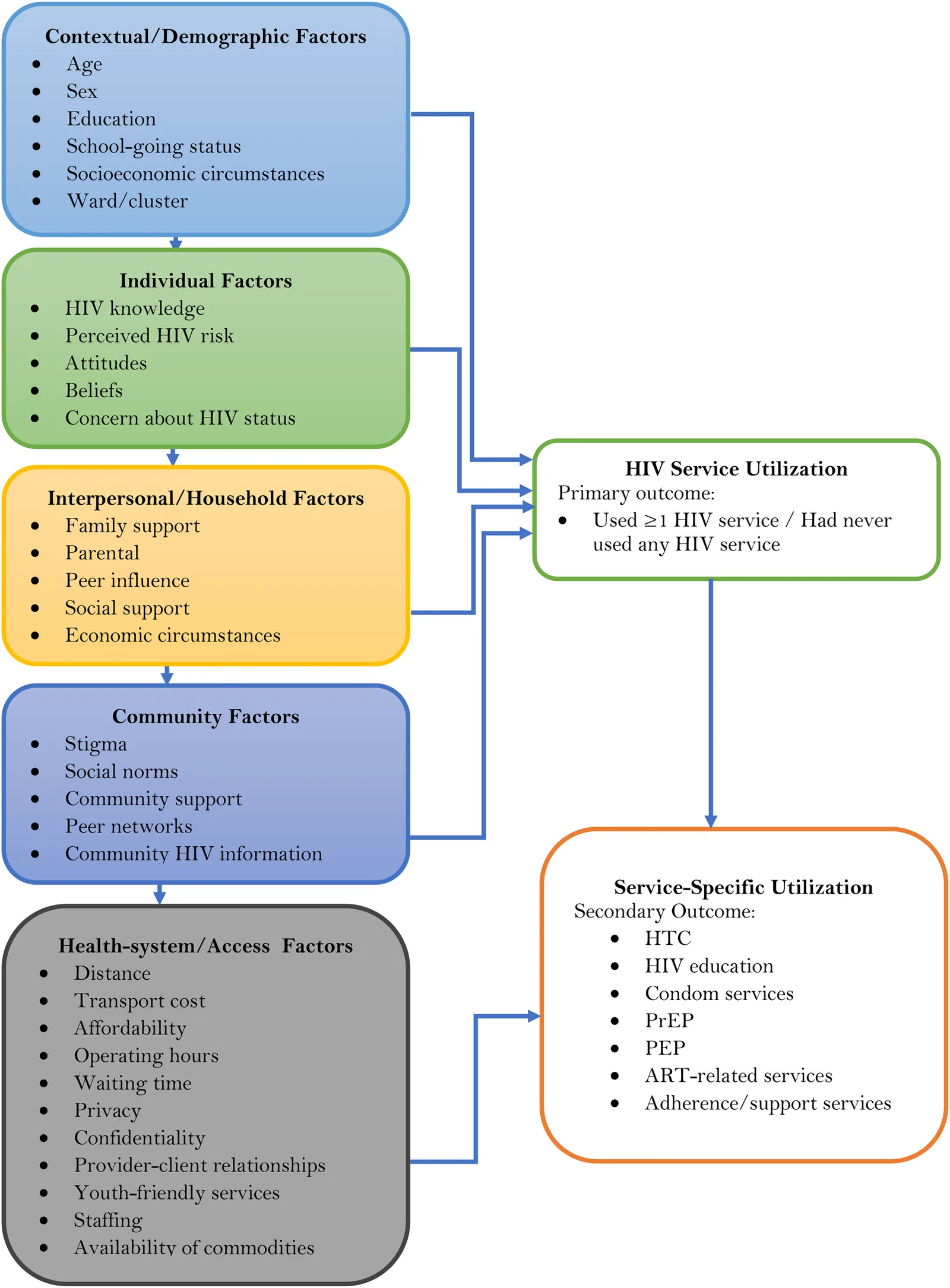
Conceptual framework on access and utilization. (Guided by the Social Ecological Model (SEM) with the WHO youth-friendly health services framework).

### Theoretical framework

#### RE-AIM

The study will use RE-AIM as a complementary implementation science framework, rather than as the primary causal framework.

The SEM will guide the identification of determinants, while the WHO youth-friendly services framework will guide assessment of service characteristics (32, 33).

RE-AIM will provide an implementation-oriented perspective. There is growing support for using the RE-AIM framework more flexibly by concentrating on only the most applicable components, rather than all five, to better suit specific research questions or stakeholder needs. The first 3 dimensions are useful in this research (34). Evaluating the application of the RE-AIM planning and evaluation framework: an updated systematic review and exploration of pragmatic application (35).

#### Reach

Reach will refer to the extent to which HIV services are accessed by the target youth population and whether particular groups experience barriers to reaching services.

Indicators will include:

- reported HIV service utilization;
- patterns of utilization across age groups;
- education and school-going status; and
- barriers affecting different youth groups.

#### Adoption

Adoption will refer to the extent to which youth-oriented HIV service arrangements are available and acceptable within the service environment.

This will include:

- availability of youth-friendly services;
- provider responsiveness;
- availability of dedicated youth service arrangements;
- peer educator involvement; and
- perceived organizational support for youth services.

#### Effectiveness

The study will not claim to measure intervention effectiveness in the causal or experimental sense, because it is a cross-sectional observational study and does not evaluate a specific intervention over time.

Participant experiences and perceptions, including satisfaction or perceived quality, will instead be treated as service-experience indicators.

#### Implementation and maintenance

Implementation and Maintenance will not be directly measured because the study does not evaluate the fidelity or long-term sustainability of a specific intervention.

### Study design

A cross-sectional mixed-methods design will be employed. The quantitative component will estimate the level and patterns of HIV service utilization among young people and examine associations between utilization and individual, household, community, and health-system factors. The qualitative component will explore young people's experiences, perceptions, barriers, and facilitators related to accessing and utilizing HIV services, thereby providing contextual explanations for the quantitative findings. The quantitative and qualitative findings will be integrated during interpretation to provide a comprehensive understanding of factors influencing HIV service access and utilization among young people.

### Study setting

The study will be conducted in Homa Bay Town Sub-County, Homa Bay County, Kenya.

Homa Bay County is characterized by a high HIV burden and substantial HIV-related vulnerability among young people. The study setting provides an appropriate context for examining the interaction between individual, social and health-system determinants of HIV service utilization.

### Study population

The study population consists of youth ages 15–24 residing in Homa Bay Town Subcounty, Kenya. The qualitative component will additionally include purposively selected key informants with relevant knowledge and experience regarding youth HIV service delivery and community dynamics, including healthcare providers, peer educators, community health promoters, chiefs, religious leaders, youth leaders, and other relevant stakeholders.

### Eligibility criteria

#### Inclusion criteria

Participants will be eligible if they:

- The participant should be 15–24 years of age at the time of recruitment.
- Should be a resident of Homa Bay Town Subcounty for at least 12 months.
- Individuals aged 18 years and above who provide consent; for those under 18 years, parental/guardian/legal representative consent is required along with personal assent.

#### Exclusion criteria

Participants will be excluded if they:

- Individuals who are intoxicated or seriously ill or in a condition that can prevent them to respond to the questionnaire appropriately.
- Individuals who have resided in Homa Bay Town Subcounty for less than 12 months.
- Participants who decline to provide informed consent or, if under 18 years of age, whose parents or guardians decline to provide consent, or the individual declines to assent.

### Sample size for quantitative component

The quantitative sample size will be determined based on the primary outcome of utilization of at least one HIV service among young people aged 15–24 years. The initial sample size will be calculated using Cochran's formula for estimating a population proportion (Cochran, 1977) (36):

$$n=\frac{Z^{2}\;.\;p\;.\;(1-p)}{E^{2}}$$

Where:

- = minimum required sample size;
- = standard normal deviate corresponding to a 95% confidence level = 1.96;
- = estimated proportion of young people utilizing an HIV-related service = 0.374 (37);
- = proportion not utilizing the service = 0.626; and
- = desired margin of erro*r* = 0.05.

Substituting the values:

$$n=\frac{1.96^{2}\;x\;0.374\;x\;(1-0.374)}{0.05^{2}}\;\;\approx \;360$$

Therefore, the minimum base sample size will be 360 participants.

A 10% non-response allowance will be considered during recruitment. The required recruitment target will therefore be:

$$n_{adjusted}=\;\frac{360}{1\;-\;0.10}\;=\;400$$

Thus, approximately 400 participants will be approached to obtain 360 completed questionnaires.

Because the study will use multistage and clustered sampling, the sampling structure will be considered during analysis when estimating standard errors and associations. A design effect will not be applied retrospectively because reliable estimates of the intracluster correlation coefficient and average cluster size will not be available.

The final quantitative sample will therefore comprise 360 complete and eligible questionnaires.

### Sample size for qualitative component

#### Focus group discussions (FGDs)

The FGDs will involve youths (male and female) selected through purposive sampling to ensure diversity in age, gender, and location. Each focus group discussion (FGD) will comprise 6–10 participants, with 1–2 FGDs conducted depending on participant diversity and the point of data saturation.

#### Key informant interviews (KIIs)

Key Informant Interviews will target individuals with critical insights into youth health, behavior, and community dynamics. Participants will be selected purposively from the following categories:

### Sampling technique

The study will employ a multistage sampling approach for the quantitative phase and purposive sampling for the qualitative phase.

#### Quantitative component

**Stage 1: Stratification**

The study area will first be stratified by ward to ensure geographical representation of the study population.

**Stage 2: Selection of Clusters**

Schools, churches, and community-based organizations (CBOs) will constitute potential sampling clusters. These settings will provide access to different categories of young people. Schools will facilitate recruitment of school-going youth, while churches and CBOs will provide opportunities to reach out-of-school youth and those not captured within the formal education system. Clusters will be selected using probability proportional to size (PPS) based on available estimates of the youth population within the respective clusters.

**Stage 3: Selection of Participants**

Within the selected clusters, eligible young people aged 15–24 years will be identified using available membership or attendance registers where applicable, supplemented by structured enumeration. Participants will then be selected using simple random or systematic random sampling. The number of participants selected from each cluster will be proportional to the estimated eligible youth population.

#### Qualitative component

For the qualitative phase, purposive sampling will be used to select participants with relevant knowledge and experience regarding access to and utilization of HIV services among young people.

**Key Informant Interviews (KIIs):** Key informants will include healthcare providers, peer educators, community health promoters, chiefs, religious leaders, youth leaders, and other stakeholders involved in youth-focused HIV service delivery. Participants will be purposively selected based on their roles, experience, and relevance to the study objectives. Approximately 12–18 KIIs will be conducted, with the final number determined by thematic saturation.

**Focus Group Discussions (FGDs):** FGDs will be conducted among young people aged 15–24 years who will not participate in the quantitative phase. Participants will be purposively selected to ensure diversity in age, sex, location, and institutional affiliation, including both school-going and out-of-school youth. Each FGD will comprise 6–10 participants, with approximately 1–2 FGDs per ward, depending on participant diversity and achievement of thematic saturation.

### Data collection

Trained research assistants will collect quantitative data using a structured questionnaire and qualitative data using focus group discussion (FGD) and key informant interview (KII) guides. Before the main data collection, the questionnaire and qualitative guides will be pretested among a small group of youth with characteristics similar to the study population but who will not be included in the final study sample. The pretest will assess the clarity, relevance, sequencing and comprehensibility of the questions, as well as the estimated time required to complete the questionnaire. Feedback from the pretest will be used to refine the instruments before commencement of the main study.

Research assistants will receive training on the study objectives, sampling procedures, administration of the data collection tools, informed consent and assent procedures, confidentiality, privacy, management of sensitive information, appropriate communication with adolescents, recognition and management of participant distress, and referral procedures. Data collection will be conducted over a maximum period of three months or until the required sample size has been achieved.

### Participant recruitment procedure

To enhance representation of youth who may not be reached through formal educational or organizational institutions, recruitment will use multiple community entry points across the selected wards. These will include schools, religious institutions, CBOs, fishing landing sites, and other community settings where young people congregate. These settings will provide access to different categories of youth, including students and youth who are out of school or less connected to formal institutions. Religious institutions and CBOs will particularly assist in reaching youth who may not be enrolled in school or participating in formal educational programmes.

Clusters will be identified within the selected wards using schools, religious institutions and CBOs that serve substantial numbers of young people.

### Participation of minors

Youth aged 18 years and above will be provided with an informed consent form and will participate only after providing voluntary informed consent. Participants aged 15–17 years will provide written assent in addition to obtaining consent from a parent, guardian, or legally authorized representative, in accordance with the procedures approved by the relevant ethics review committee. No minor will be enrolled without the required consent and assent.

Because the study addresses HIV services and sexual and reproductive health, particular attention will be given to protecting adolescents from unintended disclosure of their participation or responses. Recruitment and consent/assent discussions will be conducted in a private setting and away from peers, teachers, parents, guardians or other persons who are not part of the research process. Where parents or guardians are involved in the consent process, research staff will avoid discussing the adolescent's responses or personal information in their presence.

Participants will be informed that participation is voluntary, that they may decline to answer any question, and that they may withdraw from the study at any time without affecting their access to health services or other benefits. Participants will not be asked to disclose their HIV status unless such information is specifically required by an approved study instrument.

### Confidentiality and privacy

Given the sensitive nature of HIV-related information, interviews will be conducted in private locations where conversations cannot be overheard by other participants, family members, teachers, community members or health-care providers. Participants will be identified using unique study identification numbers rather than names on questionnaires and analytic datasets. Names, telephone numbers or other direct identifiers, where required for administrative purposes, will be stored separately from research data.

Research assistants will sign confidentiality agreements and will be instructed not to discuss individual participants, their responses or their service-use experiences outside the research team. Group discussions will also begin with an explanation of the importance of respecting the privacy of other participants. However, participants will be informed that confidentiality in an FGD cannot be guaranteed to the same extent as in an individual interview because other participants hear the information shared during the discussion. Participants will therefore be encouraged not to disclose their own or other participants’ identities or personal information outside the discussion.

### Management of sensitive disclosures and participant distress

The research team will use a participant-centred approach when sensitive information is disclosed. Participants who become distressed during an interview or discussion will be offered an opportunity to pause, skip the question or discontinue participation without penalty. Research assistants will not provide counselling beyond their training or attempt to investigate disclosures.

A referral pathway will be established before data collection in collaboration with appropriate health and psychosocial service providers. Participants who request assistance or who require additional support will, with their permission and in accordance with the approved ethical procedures, be referred to appropriate youth-friendly health, HIV prevention, sexual and reproductive health or psychosocial services. Any disclosure indicating an immediate risk of serious harm will be managed according to applicable ethical, legal and institutional requirements and the procedures approved by the ethics review committee.

The study team will maintain a referral directory containing appropriate services and contact information, and research assistants will be trained on how and when to provide referrals while maintaining participant privacy.

### Privacy during qualitative data collection

FGDs will be organized in age- and context-appropriate groups to promote comfort and reduce the likelihood of disclosure of highly personal information. Participants will be encouraged to discuss general experiences and perceptions rather than disclose identifiable personal information. Where an issue is considered too sensitive for group discussion, participants will be offered an opportunity to discuss the matter privately with a trained member of the research team or to decline to respond.

### Data management

Quantitative data will be entered into a password-protected KoboCollect database and checked for completeness, consistency, duplicate records and data-entry errors before analysis. Each participant will be assigned a unique study identification number, and direct identifiers will be excluded from the analytical dataset wherever possible. Where a linkage file is required, it will be stored separately from the research dataset and will only be accessible to authorized members of the research team.

Electronic datasets will be password protected, and access will be restricted to authorized members of the research team. Data will be securely backed up, and any transfer of research data will be undertaken using secure methods. Hard-copy documents containing participant information, where applicable, will be stored in a secure, access-controlled location.

Qualitative audio recordings, transcripts and field notes will be stored securely and will only be accessible to authorized members of the research team. Audio recordings will be transcribed verbatim and anonymized before analysis. Names and other direct identifiers will be removed from transcripts and replaced with participant identification codes.

Given the sensitive nature of HIV-related information, research assistants and other members of the research team will be required to maintain strict confidentiality and will not disclose individual participant responses or HIV-related experiences outside the research team, except where disclosure is required under applicable ethical or legal procedures. Findings will be reported in aggregate form, and qualitative quotations will be presented without information that could reasonably identify individual participants.

Data will be retained, archived and ultimately destroyed in accordance with the requirements of the approving ethics committee and applicable institutional data-management policies.

### Data analysis

Quantitative data will be analyzed using R statistical software.

#### Descriptive analysis

Categorical variables will be summarized using frequencies and percentages. Continuous variables will be summarized using means and standard deviations where approximately normally distributed, or medians and appropriate measures of dispersion where distributions are skewed.

#### Primary outcome

The primary quantitative outcome will be HIV service utilization, defined as the reported use of at least one HIV-related service during the specified recall period.

HIV service utilization will be coded as:

- 1 = utilized at least one HIV-related service.
- 0 = did not utilize any HIV-related service.

#### Secondary outcome

Service-specific utilization, including the types of HIV services accessed and the frequency of service use, will also be summarized descriptively.

#### Bivariate analysis

Bivariate analysis will be conducted to assess associations between explanatory variables and HIV service utilization. Pearson's chi-square test will be used for categorical variables where the assumptions of the test are satisfied, while Fisher's exact test will be used where expected cell counts are small.

Continuous explanatory variables will be assessed using appropriate statistical tests based on their distribution and measurement characteristics.

#### Multivariable analysis

A multivariable logistic regression model will be fitted to identify factors independently associated with HIV service utilization.

Potential explanatory variables will include age, sex, educational level, school-going status, HIV knowledge, perceived HIV risk, stigma, distance to health facilities, affordability, facility operating hours, waiting time, privacy, confidentiality, provider friendliness and youth-friendly service characteristics.

Variable selection will be informed by the study's conceptual framework, existing empirical evidence and the potential confounding role of variables. Variables demonstrating evidence of association in bivariate analyses will be considered alongside variables identified *a priori* as theoretically important. Variable selection will therefore not rely exclusively on statistical significance.

The final model will report adjusted odds ratios (AORs), 95% confidence intervals and *p*-value. The direction, magnitude and precision of the estimated associations will be considered when interpreting the findings.

#### Assessment of multicollinearity

Multicollinearity among explanatory variables will be assessed before fitting the final multivariable model using correlation diagnostics and variance inflation factors. Variables demonstrating substantial collinearity will be reviewed and, where appropriate, combined, excluded, or otherwise handled to improve model stability while maintaining theoretical coherence.

#### Model fit and diagnostics

Model adequacy will be assessed using appropriate goodness-of-fit procedures, including the Hosmer–Lemeshow goodness-of-fit test where appropriate. Additional model diagnostics will be examined to assess influential observations and overall model performance.

#### Cluster structure

Because participants will be recruited through a multistage cluster sampling approach, the analysis will account for potential correlation among participants within the same cluster. Where the number and structure of clusters permit, cluster-robust standard errors or a multilevel logistic regression model will be considered. The final analytical approach will be selected based on the observed sampling structure and the adequacy of the data for the proposed model.

The term multivariable analysis will be used consistently throughout the manuscript.

#### Missing data

The amount, distribution and pattern of missing data will be assessed before statistical analysis. The proportion of missing observations will be reported for key study variables.

Where missingness is minimal and unlikely to materially influence the findings, complete-case analysis will be used where appropriate. Where substantial missingness is identified, the likely mechanism and potential impact of missing data on the estimates will be assessed.

Where statistically appropriate, multiple imputation may be considered for variables with substantial missingness. Sensitivity analyses may also be conducted to assess whether alternative approaches to handling missing data materially affect the study findings.

#### Qualitative data analysis

Qualitative data from FGDs and KIIs will be analyzed using thematic analysis. Audio recordings will be transcribed verbatim, and transcripts will be reviewed for accuracy and anonymized before analysis.

The analysis will involve the following stages:

- familiarization with the transcripts;
- generation of initial codes;
- development and organization of categories;
- identification of candidate themes;
- review and refinement of themes;
- definition and naming of final themes; and
- development of the final analytic narrative.

A combination of deductive and inductive coding will be employed. Deductive codes will be informed by the study objectives, the Social Ecological Model (SEM), WHO youth-friendly health service principles and the study's implementation science framing. Inductive coding will allow new concepts and issues emerging from participants’ accounts to be incorporated into the analysis.

Where more than one researcher participates in coding, an initial subset of transcripts will be independently reviewed and coded to promote consistency in interpretation. Differences in coding will be discussed and resolved through consensus. Where consensus cannot be reached, a senior member of the research team will review the discrepancy and facilitate resolution.

Qualitative data will be managed and organized using NVivo qualitative data analysis software. The software will be used to facilitate systematic coding, retrieval, organization, and comparison of transcripts and themes.

Data collection and analysis will proceed iteratively, with emerging findings informing subsequent data collection where appropriate. Qualitative data collection will continue until thematic saturation is reached, defined as the point at which additional data no longer generate substantially new themes or concepts relevant to the study objectives.

#### Mixed-methods integration

The quantitative and qualitative findings will be integrated at the interpretation stage. Quantitative analysis will establish the distribution of HIV service utilization and identify statistical associations between utilization and explanatory factors, while qualitative findings will provide contextual explanations of participants’ experiences, perceptions, barriers and facilitators.

Integration will use narrative synthesis, thematic matrices and systematic comparison of areas of convergence, complementarity and divergence between the two components.

For example, quantitative evidence of geographic or financial barriers will be interpreted alongside qualitative accounts describing transportation costs, distance and practical difficulties in reaching HIV service facilities. Similarly, quantitative findings concerning provider or service characteristics will be interpreted alongside qualitative descriptions of confidentiality, stigma, provider attitudes, privacy and youth-friendly care.

This integration will allow the study to move beyond identifying statistical associations and provide a more comprehensive explanation of how individual, interpersonal, community and health-system environments shape HIV service utilization among young people.

## Data Availability

The original contributions presented in the study are included in the article/Supplementary Material, further inquiries can be directed to the corresponding author.

## References

[1] UNICEF DATA. Adolescent HIV treatment. UNICEF DATA. Available online at: https://data.unicef.org/topic/hivaids/adolescent-hiv-treatment/ (Accessed August 24, 2026)

[2] Bedingar E Bedingar N Sudfeld C. Adolescents and young adults dating and HIV perceptions: a phenomenological study in N’Djamena, Chad. Glob Public Health. (2025) 20(1):2534619. 10.1080/17441692.2025.2534619 40686401 PMC12667022

[3] Mbabazi I Abila DB Muhindo R Sekaggya C Zawedde-Muyanja S. Correlates of unawareness of HIV status and recency of HIV infections among women living with HIV: findings from population-based surveys in 13 African countries. BMJ Public Health. (2025) 3(2):e002211. 10.1136/bmjph-2024-002211 40959836 PMC12434748

[4] Ma P Shoki R Su X Ota E. Implementation strategies to promote linkage to care for key populations after HIV self-testing: a scoping review. J Glob Health Rep. (2022) 6:e55764. 10.29392/001c.55764

[5] Mashamba L Letswalo L Mkhonto F Ramalepa T. Barriers to the provision of youth-friendly services for adolescents living with HIV and AIDS. IJRBS. (2024) 13(9):177–85. 10.20525/ijrbs.v13i9.3806

[6] Staveteig S Croft TN Kampa KT Head SK. Reaching the ‘first 90’: gaps in coverage of HIV testing among people living with HIV in 16 African countries. PLoS One. (2017) 12(10):e0186316. 10.1371/journal.pone.0186316 29023510 PMC5638499

[7] Cheruiyot C Magu D Mburugu P Sagwe D. Uptake and utilization of institutional voluntary HIV testing and counseling services among students aged 18–24 in Kenya's Public universities. Afr Health Sci. (2020) 19(4):3190–9. 10.4314/ahs.v19i4.42 PMC7040318 32127896

[8] Lynn T. Together Towards an HIV-Free Kenya: Counties and Partners Reaffirm Commitment to Efficiency, Prioritization, and Collective Action. National Syndemic Diseases Control Council. (2025). Available online at: https://nsdcc.go.ke/together-towards-an-hiv-free-kenya-counties-and-partners-reaffirm-commitment-to-efficiency-prioritization-and-collective-action/ (Accessed August 24, 2026)

[9] Masaba RO Herrera N Siamba S Ouma M Okal C Mayi A. Advanced HIV disease in Homa Bay county, Kenya: characteristics of newly-diagnosed and antiretroviral therapy-experienced clients. Medicine (Baltimore). (2023) 102(51):e36716. 10.1097/MD.0000000000036716 38134082 PMC10735128

[10] Kenya Data & Statistics. HIV and AIDS in Kenya by County—Prevalence, New Infections, Deaths. Available online at: https://statskenya.co.ke/at-stats-kenya/about/hiv-in-kenya-by-county-prevalence-new-infections-deaths/109/ (Accessed August 24, 2026)

[11] Wilbourn B Howard-Howell T Castel A D'Angelo L Trexler C Carr R. Barriers and facilitators to HIV testing among adolescents and young adults in Washington, district of Columbia: formative research to inform the development of an mHealth intervention. JMIR Form Res. (2022) 6(3):e29196. 10.2196/29196 35275083 PMC8956991

[12] Wilson K Onyango A Mugo C Guthrie B Slyker J Richardson B. Kenyan HIV clinics with youth-friendly services and trained providers have a higher prevalence of viral suppression among adolescents and young adults: results from an observational study. J Assoc Nurses AIDS Care. (2022) 33(1):45–53. 10.1097/JNC.0000000000000302 34939987 PMC10329499

[13] Yu Q Zhao H Sun D Liu Y Yu Y. Prevalence and factors influencing HIV testing behavior in adolescents and young adults: a systematic review and meta-analysis. Prev Med Rep. (2025) 57:103211. 10.1016/j.pmedr.2025.103211 40838179 PMC12362408

[14] Ating’ah LO Odek A Ochieng D. Socioeconomic determinants of household access to HIV/AIDS healthcare in kibra sub-county, Nairobi county, Kenya. AJERNET. (2024) 5(4):240–52. 10.51867/ajernet.5.4.20

[15] Phiri MM Schaap A Hensen B Sigande L Simuyaba M Mwenge L. The impact of an innovative community-based peer-led intervention on uptake and coverage of sexual and reproductive health services among adolescents and young people 15–24 years old: results from the yathu yathu cluster randomised trial. BMC Public Health. (2024) 24(1):1424. 10.1186/s12889-024-18894-z 38807091 PMC11134624

[16] UNAIDS. Integrating HIV and other health services—AIDS 2020. (2020). Available online at: https://aids2020.unaids.org/chapter/chapter-5-sustainable-people-centred-approaches/integrating-hiv-and-other-health-services/ (Accessed August 25, 2026)

[17] Akatukwasa C Bajunirwe F Nuwamanya S Kansime N Aheebwe E Tamwesigire IK. Integration of HIV-sexual reproductive health services for young people and the barriers at public health facilities in mbarara municipality, southwestern Uganda: a qualitative assessment. Int J Reprod Med. (2019) 2019:1–11. 10.1155/2019/6725432 PMC6481132 31093493

[18] Embleton L Shah P Gayapersad A Kiptui R Ayuku D Wachira J. Recommendations for improving access to healthcare for street-connected children and youth in Kenya: a qualitative study. Child Youth Serv Rev. (2021) 131:106302. 10.1016/j.childyouth.2021.106302

[19] Bedingar E Paningar F Bedingar N Mbaidoum E Ngaradoum N Atun R. Rethinking HIV care for youth: insights from qualitative research with youth in Chad. Nieuwoudt, SJ, editor. PLoS One. (2025) 20(6):e0309497. 10.1371/journal.pone.0309497 40554476 PMC12186976

[20] Dzinamarira T Moyo E. Adolescents and young people in sub-saharan Africa: overcoming challenges and seizing opportunities to achieve HIV epidemic control. Front Public Health. (2024) 12:1321068. 10.3389/fpubh.2024.1321068 38566795 PMC10985137

[21] Chen-Charles J Davey J Toska D Seeley E Bekker J G L. PrEP uptake and utilisation among adolescent girls and young women in sub-saharan Africa: a scoping review. AIDS Behav. (2025) 29(6):1876–96. 10.1007/s10461-025-04656-4 40021551 PMC12075396

[22] Ndim MCF Cho FN Tongwa DZ Kum SA Tatah CA Ngome FN. Knowledge, attitudes, stigma perceptions, and practices towards HIV testing among youths aged 18–35 years in kumba, Cameroon: a community-based cross-sectional study. BMC Public Health. (2025) 25:3061. 10.1186/s12889-025-24229-3 40993574 PMC12462330

[23] Nartey EB Babatunde S Okonta KE Kotoh AM Amoadu M Abraham SA. Prevalence and barriers to the utilization of adolescent and youth-friendly health services in Ghana: systematic review and meta-analysis. Reprod Health. (2025) 22:58. 10.1186/s12978-025-02010-4 40259401 PMC12013035

[24] UNAIDS KENYA—2024 GLOBAL AIDS UPDATE. (2024). Available online at: https://crossroads.unaids.org/story/kenya/ (Accessed August 25, 2026)

[25] Piper K Stielow S Hines-Wilson M Van Alstine E Sheerin K Modrowski C. Barriers to behavioral health treatment among youth and caregivers involved in the juvenile legal system. Evid Based Pract Child Adolesc Ment Health. (2025) 10:1–16. 10.1080/23794925.2025.2602475 41541605 PMC12803726

[26] Cabecinha MA Saunders J. HIV prevention strategies. Medicine (Baltimore). (2022) 50(4):228–33. 10.1016/j.mpmed.2022.01.007

[27] Peker Ş Topuzoğlu A Hıdıroğlu S Tanrıöver Ö. Understanding the utilization of preventive health services for non-communicable diseases: a qualitative study with community members and family physicians. BMC Prim Care. (2025) 26:261. 10.1186/s12875-025-02958-z 40841597 PMC12369141

[28] UNICEF Kenya Unintended pregnancies and HIV among adolescents and young people | UNICEF Kenya. (2020). Available online at: https://www.unicef.org/kenya/reports/unintended-pregnancies-and-hiv-among-adolescents-and-young-people-Homa-Bay (Accessed August 25, 2026)

[29] Olajubu AO Olowokere AE Naanyu V. Barriers to utilization of sexual and reproductive health services among young people in Nigeria: a qualitative exploration using the socioecological model. Glob Qual Nurs Res. (2025) 12:23333936241310186. 10.1177/23333936241310186 39758890 PMC11694304

[30] Garney W Wilson K Ajayi KV Panjwani S Love SM Flores S. Social-ecological barriers to access to healthcare for adolescents: a scoping review. Int J Environ Res Public Health. (2021) 18(8):4138. 10.3390/ijerph18084138 33919813 PMC8070789

[31] Badejo O Nöstlinger C Wouters E Laga M Okonkwo P Jwanle P. Understanding why and how youth-friendly health services improve viral load suppression among adolescents and young people living with HIV in Nigeria: realist evaluation with qualitative comparative analysis. BMJ Glob Health. (2023) 8(9):e012600. 10.1136/bmjgh-2023-012600 37748794 PMC10533666

[32] WHO. Making health services adolescent friendly. (2012). Available online at: https://www.who.int/publications/i/item/9789241503594 (Accessed September 1, 2026)

[33] Muhumuza R Ndekezi D Nagawa R Awino E Bwalya C Mbewe M. Navigating HIV self-testing: concerns among adolescents and young people aged 15–24 years in Uganda. An exploratory qualitative study. Abu-ba’are GR, editor. PLoS One. (2026) 21(2):e0330000. 10.1371/journal.pone.0330000 41642913 PMC12875464

[34] D’Lima D Soukup T Hull L. Evaluating the application of the RE-AIM planning and evaluation framework: an updated systematic review and exploration of pragmatic application. Front Public Health. (2022) 9:755738. 10.3389/fpubh.2021.755738 PMC8826088 35155336

[35] Glasgow RE Harden SM Gaglio B Rabin B Smith ML Porter GC. RE-AIM planning and evaluation framework: adapting to new science and practice with a 20-year review. Front Public Health. (2019) 7:64. 10.3389/fpubh.2019.00064 30984733 PMC6450067

[36] Nanjundeswaraswamy TS Divakar S. Determination of sample size and sampling methods in applied research. PES. (2021) 3(1):25–32. 10.24874/PES03.01.003

[37] Ayugi J Cheruiyot I Opondo P Olashore A Assessment of voluntary HIV counseling and testing service utilization and VCT/HIV related knowledge and attitude towards VCT among out of school youth in rural Nakuru County, Kenya: a cross sectional survey. (2017). 10.11604/pamj.2017.26.83.11212 PMC5410012 28491214

